# Detection of lumpy skin disease virus in cattle using real-time polymerase chain reaction and serological diagnostic assays in different governorates in Egypt in 2017

**DOI:** 10.14202/vetworld.2019.1093-1100

**Published:** 2019-07-24

**Authors:** Gamil Sayed Gamil Zeedan, Ayman Hamid Mahmoud, Abeer Mostafa Abdalhamed, Khaled Abd El-Hamid Abd El-Razik, Manal Hamdy Khafagi, Hala Abdoula Ahmed Abou Zeina

**Affiliations:** 1 Department of Parasitology and Animals Diseases, National Research Centre, 33 Bohouth St., Dokki, Giza, P.O. Box 12622, Egypt; 2 Department of Biotechnology and Food Hygiene, Animal Health Institute, Dokki, Giza, Egypt; 3 Department of Reproduction Diseases, National Research Centre, 33 Bohouth St., Dokki, Giza, P.O. Box 12622, Egypt

**Keywords:** enzyme-linked immunosorbent assay, indirect fluorescent antibody technique, lumpy skin disease, polymerase chain reaction, *Poxviridae*, real-time polymerase chain reaction

## Abstract

**Background and Aim:**

Lumpy skin disease (LSD), is a highly infectious viral disease of cattle, caused by LSD virus (LSDV) which belongs to the genus *Capripoxvirus* of family *Poxviridae*. In the summer of 2017, skin lesions suggestive of LSD were observed in cattle at several governorates in Egypt. This study aimed to detect LSDV in cattle specimens using rapid serological and molecular diagnostic assays.

**Materials and Methods:**

A total of 46 skin biopsies and uncoagulated blood samples were collected from cattle with LSD suggestive clinical signs, as well as 290 coagulated whole blood samples from cattle without skin lesion in different governorates in Egypt during the summer of 2017. Skin biopsies were used for virus isolation from the chorioallantoic membrane of 11-day-old specific pathogen-free embryonated chicken eggs (SPF-ECEs). LSDV was identified using conventional polymerase chain reaction (PCR), real-time PCR (RT-PCR), and fluorescent antibody technique (FAT) with specific hyperimmune serum against LSDV. Cattle sera were examined using indirect FAT (IFAT) and indirect enzyme-linked immunosorbent assay (ELISA).

**Results:**

Skin nodules and sitfast lesions were significant clinical signs observed in all LSD suspect cattle. SPF-ECEs, from which positive isolations were made and it showed characteristic inflammatory and focal white pock lesions. The isolated viruses were identified as LSDV by FAT, conventional gel-based PCR, and RT-PCR. Among the skin biopsies and corresponding blood samples, LSDV-positive samples percentage were 39.13 and 36.95 by RT-PCR, followed 34.78 and 28.26 by conventional PCR and then 32.6 and 26.8 by FAT, respectively. The total positive percentage of LSDV antibody detected in cattle serum samples were 17.93 and 14.48 by indirect ELISA and IFAT.

**Conclusion:**

LSDV was detected and identified in skin biopsies and corresponding blood samples of naturally infected cattle, more LSDV-positive samples were detected by RT-PCR, followed by conventional PCR and then FAT. The indirect ELISA detected more antibody-positive samples than the IFAT from cattle serum samples. The RT-PCR assay is simple, sensitive, rapid, and reliable for the detection of LSDV in blood and skin nodule biopsies of suspected cattle.

## Introduction

Lumpy skin disease (LSD) is a viral cattle disease caused by a Neethling strain LSD virus (LSDV) belonging to the genus *Capripoxvirus*, subfamily *Chordopoxvirinae*, and family *Poxviridae* [1,2]. All *Capripoxviruses* have a double-stranded DNA genome of approximately 150 to 151 kbp long [3]. LSDV is closely related to other *Capripoxviruses*, but there is a unique competent structure (encoding complement genes) that is responsible for LSDV virulence and host range [4]. LSD is one of the most important threats to beef and dairy farming in Africa and the Middle East due to skin destruction, reduced milk production, abortions, low weight gain, and secondary bacterial infections [5]. LSD signs can be the subclinical or clinical and include fever, skin nodules that cover the entire animal’s body, and edema of the limbs and brisket with lameness and enlarged of the superficial lymph node in few animals [6]. LSDV is transmitted through direct contact between infected and noninfected animals, indirect mechanical and biological transmission by *Aedes aegypti* mosquitoes, *Culicoides*, and hard *Ixodid* ticks that are associated with wet, warm summer seasons have been reported [7,8]. Cattle of all breeds, including wild ruminants, are susceptible [6,9]. LSD was first reported in Zambia in 1929, and it subsequently spread to South Africa to Southern African countries. LSD was first detected outside Africa in Palestine in 1989, followed by detection in Sinai Egypt, Bahrain, Kuwait, Oman, Yemen, Lebanon, Jordan, and Turkey [10,11]. The first diagnosis of LSD in Egyptian cattle was in the summer of 1989, followed by outbreaks in 2006, 2011, 2014, and 2017 [12-17].

In 2017, outbreaks LSDV in Egypt re-introduced of LSDV through imported cattle from Ethiopia or other endemic countries and unrestricted animals’ movement across country borders is a major and constant threat for LSDV. Furthermore, LSDV was detected in Bulgaria, Macedonia, Serbia, Albania, Montenegro, and Kazakhstan [18,19]. Controlled of LSDV can be achieved by vaccination and restrictions animal movement [20]. The diagnosis of LSD requires rapid and reliable laboratory diagnostic tools for confirmation of disease [20]. Diagnosis is performed using viral isolation in embryonated chicken eggs (ECEs), tissue culture, and agent identification methods such as fluorescent antibody technique (FAT) and polymerase chain reaction (PCR)-based assays [20,21]. Serological diagnosis of LSD employs tests such as agar gel immunodiffusion, indirect enzyme-linked immunosorbent assay (ELISA), indirect FAT (IFAT), and western immunoblotting [10,13]. The disadvantage to serological tests is that the tests cannot differentiate between infected and vaccinated animals or antibodies resulting from LSDV infection from those of other *Poxviruses* [9-11]. Molecular-based diagnosis of LSDV several PCR methods have been developed. Recently, real-time PCR (RT-PCR) is the more effective molecular method, fast, closed systems, not need any post-PCR-electrophoresis, reducing risks from contaminations, specific and highly sensitive diagnosis as well as easy to detect and analysis of mutations, including single-nucleotide polymorphisms [22].

Genotyping can, however, be performed by RT-PCR without the need for multiplexing by using melting point temperature (Tm) between the probe and its target will occur at a different Tm for each strain. The melting peaks postfluorescence melting curve analysis; serve in differentiating vaccine, virulent LSDV isolates, sheep pox (SPP), and goat poxviruses [22,23].

The present study aimed to detect LSDV in infected cattle specimens using rapid serological and molecular diagnostic assays in different Governorates in Egypt.

## Materials and Methods

### Ethical approval

All samples were collected as per standard procedure without giving any stress or harm to the animals. The work was conducted according to the European Union [24], and the guidelines of the National Institutes of Health Guide [25]. All laboratory work was conducted at the National Research Centre laboratories, Cairo, Egypt.

### Sample collection and processing

Sporadic cattle showed clinical signs suggestive of LSD in the summer of 2017 in different governorates (Beni Suef, El-Fayoum El Giza, El-Menia, El-Gharbia, El-Qalyubia, and Sharkia) in Egypt [26,27]. Whole coagulated blood samples (serum samples n-290) were collected from animals with or without clinical signs and from those vaccinated or not vaccinated with the SPP vaccine (Romanian strain, 10^3.5^ TCID 50/dose). Skin biopsies and uncoagulated blood samples (n-46) were obtained from animals with clinical signs of LSD in different governorates (Beni Suef, El-Fayoum El Giza, El-Menia, El-Gharbia, El-Qalyubia, and Sharkia) in Egypt. Each biopsy sample from a clinical case was ground using a sterile mortar and pestle and suspended in phosphate-buffered saline (PBS) containing 10% antibiotic solution. Each tissue homogenate was centrifuged at 3000 r.p.m for 10 min at 4°C. The clear supernatant fluid was collected, frozen at −20°C, thawed 3 times, and centrifuged at 3000 rpm for 15 min. A volume of 2 ml of the supernatant was collected in sterile tubes and stored at −20°C until used. Whole blood samples without anticoagulant were collected from all animals with or without LSD signs. After collection, whole blood was allowed to clot by leaving at 37°C for 15-30 min, centrifuging at 3000 r.p.m for 10 min, immediately transferring the serum into 0.5 ml aliquots and storing at –20°C.

### References virus strain and positive LSDV antiserum

The reference LSDV strain (Neethling strain), sheep vaccine against the Romanian strain (10^3.5^ TCID 50/dose), and positive LSDV antiserum were kindly provided by the Animal Health Research Institute, Dokki, Giza, Egypt.

### Virus isolation and titration

Skin biopsy samples previously prepared were inoculated into the chorioallantoic membrane (CAM) of 9-11-day-old eggs using the specific pathogen-free ECE (SPF-ECE) route for three blind passages according to the method described by the OIE [25]. Briefly, 200 μl from the supernatant fluid of each tissue homogenate was inoculated into the CAM of 5 chicken embryos, incubated at 37°C, and examined daily. The positive CAMs were harvested, homogenized, and minced using a pestle and mortar, and centrifuged at 3000 rpm for 10 min in a cooling centrifuge at 4°C. The supernatant fluids were kept at −20°C for identification using FAT and PCR. The isolated LSD-virus stock was titrated in ECE with a virus titer of 10^9^ EID/50 in 1 ml according to the method by Reed and Munch [28].

### Detection of LSDV antibodies

#### IFAT test

The indirect IFAT was used to detect serum antibody against LSD. The 290 serum samples were prepared for the IFAT test. The antigen used to detect the serum antibody against LSD. A volume of 50 µl of 100 TCID 50 of reference LSDV strain (Neethling strain), per 10 circles in flat glass slides after dropped fixed by cold acetone at −20°C for 30 min. The diluted 1/25 tested serum was flooded on each circle on glass slides, then incubate in humid chamber at 37°C for 60 min and washed 3 successive times in PBS pH 7.2 (PBS) allowed to dry, blocking each glass slide blocked by blocking buffer (0.5% fetal bovine serum in PBS for blocking of nonspecific background reaction. The positive and negative control sera were also included in the glass slide. 50 ml of diluted rabbit anti-Bovine gamma-globulin (immunoglobulin G [IgG]) conjugated fluorescent isothiocyanate was added to each circle and incubated in humid chamber at 37°C for 30 min and washed 3 successive times in PBS pH 7.2 (PBS) allowed to dry. The prepared glass slides wet under coverslips with Tris-buffered glycerol pH 9 examined by fluorescent microscope under 40×. The positive test showed bright fluorescence foci where the antibody reacted with the virus, and the negative serum showed a dark field or oblique green foci.

#### Indirect ELISA

Antibodies against LSDV were detected in the collected serum samples using ELISA, according to Bhanuprakash *et al*. [2]. The supernatant of LSDV-infected CAM of SPF-ECE was centrifuged at 3000 rpm for 20 min, and the pellet was sonicated and clarified. The soluble LSDV proteins were collected and used according to checkerboard titrations of ELISA. Microtiter-plates (Nunc, Denmark) was coated with sonicated LSDV antigen diluted 1:100 in 0.05 M carbonate buffers, pH 9.6 and incubated overnight at 4°C. The plate was washed 3 successive times with washing buffer (PBS, pH 7.2 containing 0.05% tween-20 (PBST). Added 100 µl of blocking solution contains 1% bovine serum album incubated for 1 h at 37°C and the plates were washed 3 times with PBST. Added 100 µl of diluted tested serum samples 1:50 in PBS; control positive and negative serum were included for 1 h at 37°C; and the plates were washed 3 times with PBST. 100 ml of conjugated anti-bovine horseradish peroxidase diluted at of 1:3000 in PBST according to the manufacturer’s instructions, the plates were then incubated for 1 h at 37°C after incubation the plates were washing 3 successive times in PBST. A volume of 100 µl of O-Phenylenediamine substrate solution was added and incubated for 10-15 min incubated at room Tm in dark places thereafter the reaction was stopped by adding 50 µl of 2 M H_2_SO_4_. The ELISA plate was read at a wavelength of 492 nm using an ELISA reader. The cutoff value used was higher than the mean of optical density values of control negative sera.

#### Viral DNA extraction

DNA was extracted from skin biopsy homogenate and blood samples using QIAamp DNA Mini Extraction Kit (Qiagen, Germany) and DNA extraction kits (iNtRON, Korea^#^Cat No. 17154) according to the manufacturer’s instructions. The reference LSDV strain was used as a positive control, and sterile deionized water was used as a negative control. In all cases, DNA was eluted in 100 μl of elution buffer and stored at −20°C until further analysis.

#### Conventional PCR

The PCR procedures were performed according to Sambrook *et al*. [29]. The primer sets developed from the gene of viral attachment protein gene, VP32 was used. Forward primer, 5′-TTTCCTGATTTTTCTTACTAT-3′ and reverse primer, 5′-AAATTATATA C GTAAATAAC-3′ designed by Ireland and Binepal [30]. Reactions volumes of 25 µl containing 0.2 µl each of forward and reverse primers at a final concentration of 20 pmol of each primer (2.5 units), 12.5 µl master mix, 2.5 µl of DNA template (contains 200 ng) and nuclease-free sterile double distilled water up to 25 µl. Negative and positive controls were included for each reaction. Amplification was done under the following conditions: Initial denaturation cycle at 95°C for 2 min, 40 cycles (denaturation at 95°C for 30 s, annealing at 55°C for 30 s, and extension at 72°C for 1 min), followed by a final extension cycle at 72°C for 10 min. Amplicons (5 µl) were separated on a 1.5% agarose gel at 100 V for 30 min according to the methods described by Ireland and Binepal [30]. The specific primers set amplified a DNA fragment of 192 bp equal to the expected amplification product size from LSDV. The DNA Ladder 100 bp (GeneDireX^®^ Inc., USA) was used as a size standard, and the gel was visualized using an ultraviolet light transilluminator.

#### RT-PCR

The test was carried out according to the method of Dejan *et al*. [31]. LSDV DNA amplification was done using RT-qPCR1oo reaction kits (GPS, Alicante, Spain code #1013046#) contains a dehydrated mixture of specific primers and labeled probe, dNTPs, additional internal control primers, probe and DNA template and slandered template; 2.4×10^7^ target copies for positive control. qPCR was done in a reaction 10 µl volume containing 2 µl of the qPCR master mix, 0.5 µl of primers and probe mix with the reference dye FAM and ROX, 2.5 µl of DNA template, and fill up to 10 µl with distilled DNase and RNase-free water. The optimized cycle program for RT-PCR as the following thermal cycles conditions were used: 95°C for 15 min, followed by 40 cycles in 2 steps: (a) 95°C for 15 s (denaturation) and (b) 60°C for 60 s (combined annealing/extension). The fluorescence levels were measured at the end of each cycle. The analytical method was determined by using RT System (Bio-Rad, USA). Analysis of fluorescence data was performed using the CFX Software (Bio-Rad Laboratories).

#### FAT

FAT was used to detect LSDV in CAM and skin biopsies suspension according to OIE [27] as follows: 50 µl of each 46 skin biopsy samples and infected CAM with pock lesion suspension were transferred to circles in glass slides. The positive LSDV and negative control were included in glass slides. These slides were left to dry in air for 30 min and then fixed by cold acetone at −20°C for 30 min and washed 3 successive times in PBS pH 7.6 and then allowed to dry, then blocked each glass slide by blocking buffer (0.5% fetal calf serum [FCS] in PBS) for blocking of nonspecific background reaction. Washed by PBS pH 7.6 and then added 50 µl 1:100 diluted Rabbit hyperimmune serum and kept at the humidified chamber at 37°C and then washed 3 successive times and blocked with 0.5 FCS in PBS at pH 7.6. 50 µl of diluted anti-rabbit gamma-globulin (IgG) conjugated fluorescein isothiocyanate was added to each circle and incubated in humid chamber at 37°C for 60 min and washed 3 successive times in PBS pH 7.2 (PBS) then allowed to dry. The prepared glass slides were mounted with 50% Tris-buffered glycerol pH 9 and covered with a coverslip and examined by fluorescent microscope under 40×. The positive test showed bright fluorescence foci where the antibody reacted with the virus, and the negative serum showed a dark field or oblique green foci.

### Statistical analysis

Statistical analysis for various serological and molecular methods by using percentage and Fisher’s exact test at 95% of confident interval and (p≤0.05) used the Statistical Package for the Social Sciences (SPSS, version 16 (Chicago, Illinois, USA).

## Results

**Figure-1:**
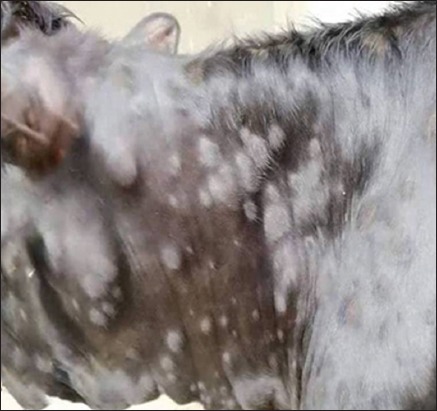
A calf infected with lumpy skin disease virus showing sitfast lesions characterized by large necrotic skin nodules.

All suspected cases in different governorates (Beni Suef, El-Fayoum El Giza, El-Menia, El-Gharbia, El-Qalyubia, and Sharkia) in Egypt showed clinical signs of LSD. The clinical signs in affected cattle included increased body Tm (40-41°C), depression, inappetence, loss of appetite, salivation, naso-ocular discharge, pneumonia, corneal opacity, severe shoulder edema, brisket, and circumscribed nodules on the skin of different sizes that cover the entire animal’s skin including the head, neck, trunk, perineum, udder, and teats. Most of the necrotic nodules were ulcerations and formed deep scabs called sitfast (Figure-1).

### Detection of LSDV antibody using ELISA and IFAT

**Figure-2:**
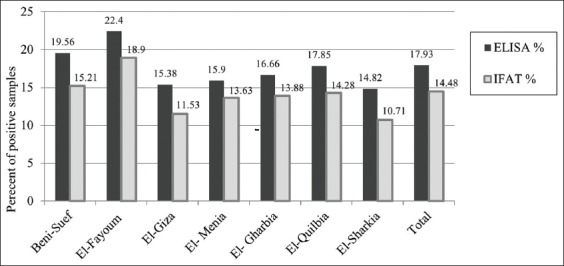
Detection of antibody against lumpy skin disease virus in cattle sera by enzyme-linked immunosorbent assay and indirect fluorescent-antibody technique in different governorates of Egypt.

Positive results were obtained in 52 out of 290 serum samples tested by ELISA (17.43%). Of 290 serum samples tested by IFAT, 42 showed positive results (14.42%). The highest positive percentage among serum samples collected came from cattle examined by IFAT and ELISA for El-Fayoum (18.9% and 22.4%) followed by Beni Suef governorate (15.21 and 19.56%). Total positive sera against LSDV in different Egyptian governorates were 14.48% and 17.93% based on indirect IFAT and IELISA, respectively (Figure-2).

### Isolation and identification of LSDV

**Figure-3:**
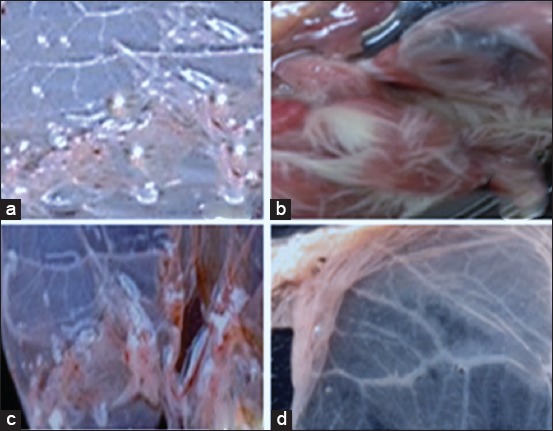
(a and c) The characteristic pock lesion of lumpy skin disease virus on chorioallantoic membrane of specific pathogen-free embryonated chicken eggs after third passages. Furthermore, the embryo showed inflammatory signs, as (b and d), depicting the negative control chorioallantoic membrane, showed no inflammatory signs or pock lesions.

LSDV was isolated from skin biopsy homogenates collected from infected cows (n-18) based on the appearance of characteristic pock lesions on the CAM of ECE (Figure-3). The LSDV isolates were identified using RT-PCR, conventional gel-based PCR, and FAT.

### Conventional gel-based PCR

**Figure-4:**
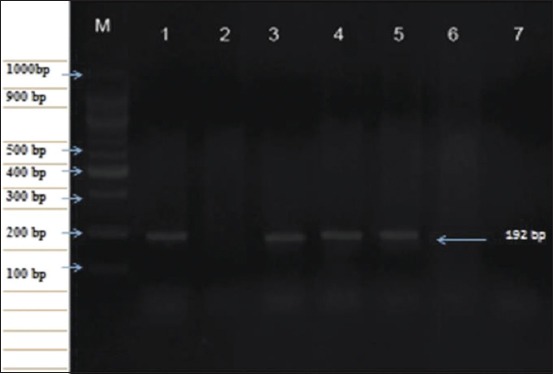
Polymerase chain reaction amplicon of lumpy skin disease virus (LSDV) genomic DNA was detected in skin biopsies and blood in EDTA compared to the reference strain of LSDV using specific gene (P32) viral attachment gene at 192 bp on a 1.5% agarose gel. Lane: M, 100 bp DNA ladder; L M: 100 bp DNA ladder, Lane 1: Positive control (references LSDV); Lane 2: Negative control, Lane 3-5: Positive samples from skin biopsy or blood in EDTA; Lane 6-7: Negative samples.

LSDV was detected using PCR with a specific primer set amplifying a 192 bp DNA fragment equal to the expected amplification product size from LSDV. The reference strain of the LSDV-infected CAM, skin biopsies, and blood in EDTA were collected from suspected cows in different Egyptian governorates. Viral LSDV DNA was detected in 16 (out of 46) skin biopsies collected from cows by using PCR and 13 (out of 46) blood samples with EDTA. The amplicon size of the PCR product in positive samples had a molecular weight of 192 bp for the attachment protein gene equal to the expected amplification product size from the reference LSDV. Local CAM isolates and skin nodules biopsies samples showed no differences between the strains (Figure-4).

### RT-PCR

The presence of specific Ct curves of DNA templates extracted from CAM with pock lesion, skin biopsy, and its related blood samples from infected cattle more than threshold curve nearly similar to Ct of DNA template positive control of LSDV. The positive percent of RT qPCR for skin biopsy and its related blood samples from infected cattle were 39.13% and 36.95%, respectively.

### FAT

FAT was adapted for the identification of LSDV protein in infected positive CAM with pock lesion of isolated virus and in skin lesion suspension collected from infected cattle using specific hyperimmune serum against LSDV, specific yellowish green fluorescent granules emitted from positive suspension. The percent of positive LSDV in blood and skin biopsy samples was 32.6% and 26.08%, respectively.

The antigen detected by different diagnostic methods were not significantly different (p>0.005).

Sensitivity = [TP/(TP+FN)] × 100,

where TP = True positive and FN = False-negative

Specificity = [TN/(TN+FP)] × 100,

where TN = True negative, FP = False positive result

Accuracy = True positive + True negative/Total number of samples × 100

The sensitivity of RT-PCR, PCR, and FAT were evaluated on 46 samples from naturally infected cattle, skin biopsy lesion (n-46), and cattle whole blood (n-46. The summarized results of RT-PCR detected the highest percent of LSDV in both blood and skin biopsy samples were 36.95% and 39.13%, followed by gel-based PCR were 28.26% and 34.78%, and the lowest detection were 32.6% and 26.08% in blood and skin biopsy samples by FAT accordingly (Table-1).

**Table 1:** Comparison between different assays for detecting LSDV from skin biopsies and whole blood collected from cattle.

Type of sample	n	Conventional PCR		Real-time PCR		FAT	
		
		+	%	+	%	+	%
Skin biopsy	46	16	34.78	18	39.13	15	32.6
Blood	46	13	28.26	17	36.95	12	26.08

+: Positive results; %: Percent. LSDV: Lumpy skin disease virus

## Discussion

**Figure-5:**
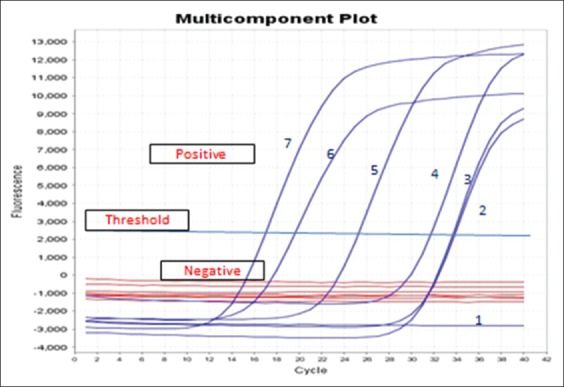
Amplification plot of real-time polymerase chain reaction showed positive results for curve numbers 2, 3, 5, and 6 for DNA extracted from field samples skin biopsy and its related blood of infected cattle before virus isolation. Curve 7: DNA from positive chorioallantoic membrane with pock lesion of the isolated virus. Curve 4: Ct of DNA template positive control of lumpy skin disease virus. Curve 1: DNA-free sample negative control (graph generated by ES Equant tube scanner software).

LSDV is a highly contagious viral cattle disease that causes economic losses [32]. LSD is endemic to several regions of Africa and the Middle East, including Egypt, Palestine, Iraq, Iran, Lebanon, and Jordan. Recently, it was transmitted to Europe in Turkey, Cyprus, and Greece [33,34]. LSD in cattle is characterized by the sudden appearance and rapid spread of lumps on the skin after a fever. The nodular skin lesions are distributed along the skin of animals along with internal organs, fever, poor growth, and lymphadenopathy [34]. Mechanical transmission of LSDV between different cattle breeds by hematophagous arthropod vectors such as mosquitoes and stable flies [35]. Direct contact between cattle or contacts through milking procedure were also reported as potential transmission modes. Although sheep, goats, and water buffalo are in contact with infected cattle during the LSD outbreak, they remained clinically healthy [34]. SPP, goat pox (GTP), and cowpox share common major antigens for neutralizing antibodies; hence, it is difficult to distinguish between them using serological tests. However, LSD control depends on a rapid and accurate diagnosis [36]. Clinical LSD signs were found in cattle in different Egyptian governorates during the summer of 2017. Viruses were isolated on CAM, and viral isolates were identified using RT and gel-based PCR to detect LSDV-DNA in clinical samples (skin biopsies and blood in EDTA). All assays confirmed that the outbreaks were caused by LSDV, and those results were supported by many authors [36-38]. Clinical LSD signs characterizing LSD outbreak included skin nodules all over the body, including the head and neck (Figure-1), which was similar to LSD lesions recorded in previous outbreaks [33,34]. Surprisingly, LSD infection was higher in adult cattle than in young calves. The equal exposure to LSDV suggested that the possibility of lower prevalence in calves than adults’ cattle population may be due to fewer insects in their shelter or passive maternal immunity that protects calves against LSDV. This observation agrees with a previous study [38] reporting that SPP virus vaccine could not protect Egyptian cattle against LSD and that 5% of cattle vaccinated with live attenuated sheep and GTP vaccine (Kenya strain, KS1) developed clinical signs of LSD. In Egypt during the summer of 2017, LSD was observed in cattle previously vaccinated by Romanian’s SPPV vaccine. This occurred previously in Ethiopia due to the lack of LSDV antibody protection. This may be due to lack of cross-protection leading to clinical disease in vaccinated cattle [39]. Serological assays are useful in screening the prevalence of LSDV, but they are too time-consuming to be used as primary diagnostic tools. Serum sample testing with LSDV antibodies may be difficult due to the cross-reactivity encountered with other poxviruses and the low antibody titers elicited in some animals following mild infection or vaccination [40]. The present study showed that the percentage of positive serum samples collected from cattle during LSD outbreak tested by ELISA and IFAT was 17.93% and 14.48%, respectively (Figure-2). These results can be explained by differences between serological assays for the detection of LSDV antibodies in cattle sera may be due to the differences in sensitivity of serological assays or may be due to cross-reactivity encountered with other *Capripoxviruses* as well as to the low antibody titers elicited in some animals following mild infection or vaccination. LSDV was isolated from skin nodule of infected cattle on CAM of SPF-ECE with observed pock lesions and mild inflammatory signs on the embryos after the first passage and become clear after the third passage (Figure-3), and this result agrees with [39], with further identification by FAT in addition to, molecular characterization of virus isolate using PCR with primers specific to the attachment gene (192-bp) such that the PCR product could be used to detect LSDV in skin biopsy, blood, and harvested CAMs of ECE (Figure-4) due to successive targeted the LSDV envelope protein-like gene to amplify the specific products from the extracted DNA products and the bands were clear and sharp by increasing the DNA concentration from target and were sensitive to detect LSDV strain in its original skin samples and their resource and this result agrees with previous results [25]. Detection of the DNA fragment in whole blood and skin biopsy samples by RT-PCR more than conventional PCR may be due to collect samples from animals later in the disease course; the presence of the virus in blood was at a low level or there was low sensitivity for conventional PCR in detecting LSDV DNA in blood (Figure-4 and Table-1) [40]. They found that the RT-PCR assays detected LSDV-DNA in biopsy samples with the lowest Ct values, making them most suitable for testing. This study showed that the RT-PCR detected the highest percent of LSDV in both blood and skin biopsy samples were 36.95% and 39.13%, followed by gel-based PCR were 28.26% and 34.78%, respectively, (Figure-5 and Table-1) and this result can be explained by conventional PCR could not detect low viral DNA copy load. RT-PCR can detect low amounts of viral genomes in biopsy and blood samples. This result is agreed with [41-44]. Finally, the RT-PCR was rapid and accurate than conventional PCR for detecting LSD viral genomes in specimens collected from infected cattle.

## Conclusion

LSDV antibodies were detected in cattle in different Egyptian governorates during the summer of 2017 using indirect (ELISA and IFAT). LSDV was isolated from skin biopsy collected from clinically suspected cattle on CAM of ECE-SPF. The identification of LSDV isolates from a skin biopsy, blood samples by conventional PCR, RT-PCR, and FAT. RT-PCR assays are simple, accurate, and rapid can replace conventional PCR for detecting LSDV-DNA in clinically infected cattle specimens.

## Authors’ Contributions

GSGZ, AMA and AHM: designed the study, performed the laboratory work (PCR and RT-PCR) and drafted the manuscript. KAA, MHK and HAAA: Shared in the conception of the research idea, sharing laboratory work, provided some reagents and materials and helped in manuscript preparation. All authors have read and approved the final manuscript.
